# Supplementary material for: Replication intermediates that escape Dna2 activity are processed by Holliday junction resolvase Yen1
Source: Nat Commun. 2016 Oct 25;7:13157. doi: 10.1038/ncomms13157 (PMC5093310; doi:10.1038/ncomms13157)
Supplement: Supplementary Information — Supplementary Figures 1-9, Supplementary Table 1 and Supplementary Reference. [file ncomms13157-s1.pdf]

Supplementary Information

Supplementary Figures

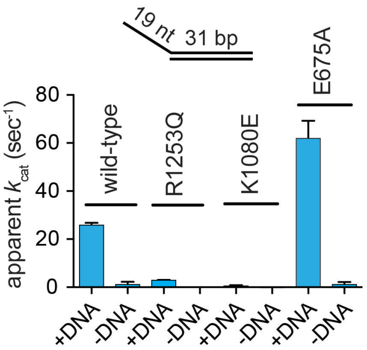

Supplementary Figure 1 │ Apparent ATP turnover number of Dna2 proteins. Apparent k_{cat} values were calculated from the initial rate of ATP hydrolysis in experiments such as those shown in Fig. 1 c, containing a 5'-tailed DNA substrate, but with 3 nM Dna2 variants. Data presented as mean values ±s.e.m. (n = 2).

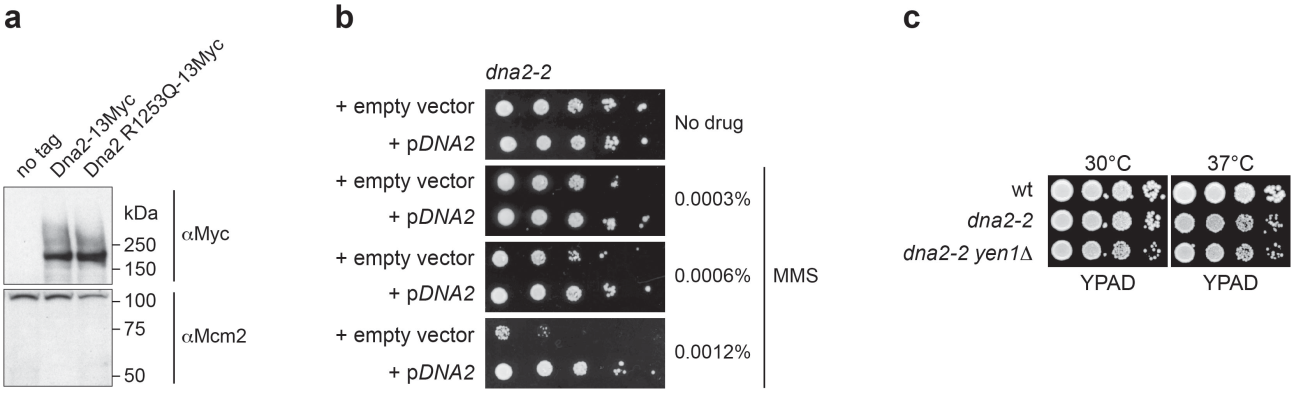

Supplementary Figure 2 │Assessment of Dna2 helicase-defective *dna2-2* cells. (**a**) Western blot analysis showing that the R1253Q mutation within the helicase domain of Dna2 does not alter protein expression or protein stability. Whole cell extracts were prepared from logarithmically growing cultures and resolved on a NuPAGE 7% Tris-acetate gel (Life Technologies). The upper part of the membrane was probed for wild-type and mutant Dna2 tagged with 13 x Myc at the C-terminus and expressed from the endogenous *DNA2* locus. The lower part of the membrane was probed for Mcm2, which served as loading control. The positions of size-markers are indicated. (**b**) Drop assay, performed as described for Fig. 3, showing that *dna2-2* cells are sensitive to MMS, and that expression of *DNA2* from a low-copy number plasmid with a GPD promoter restores resistance. (**c**) Drop assay showing that *dna2-2 yen1*Δ cells are viable at elevated temperature.

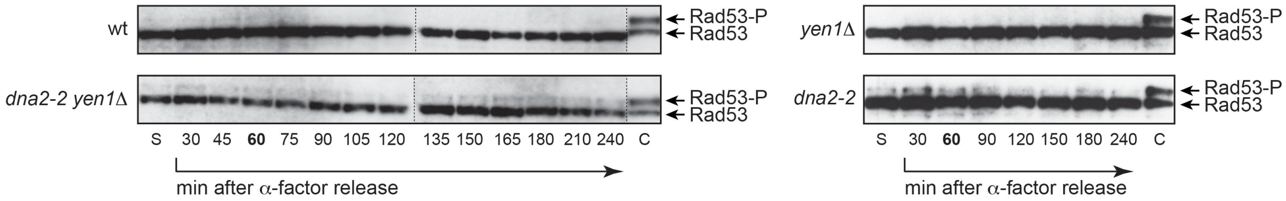

Supplementary Figure 3 │Unscheduled post-replicative checkpoint activation in Dna2 helicase-defective cells is not discernible in unperturbed conditions. The indicated strains were synchronized in G1, released into YPAD medium without HU, and monitored by Western blot analysis for phosphorylation of Rad53 over a period of 4 h. Under these conditions, cells routinely completed bulk DNA synthesis within 60 min of α-factor release (see Fig. 2c). S, synchronous; C, control samples showing Rad53 phosphorylation (Rad53-P) after exposure of the respective strains to HU.

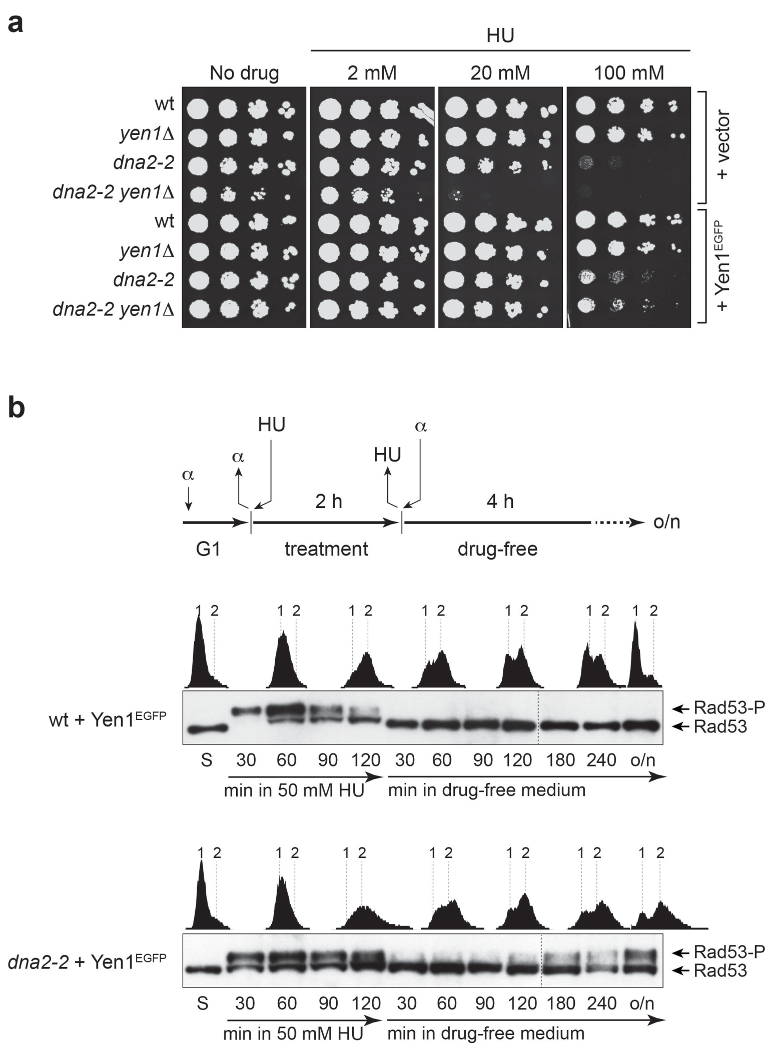

Supplementary Figure 4 │Green fluorescent protein-tagged Yen1-EGFP is functional. (**a**) Drop assay, performed as described for Fig. 3, showing that Yen1-EGFP suppresses the severe HU sensitivity of *dna2-2 yen1*Δ cells, which demonstrates that the tag does not interfere with Yen1 function. (**b**) Biphasic checkpoint activation in response to acute replication stress is maintained in Dna2 helicase-defective cells expressing Yen1-EGFP. Mitotic time-course experiments performed as described for Fig. 4. Checkpoint activation and replication progression were monitored by analyzing Rad53 phosphorylation (Rad53-P) and DNA content (1N and 2N indicated), respectively. S, synchronous; o/n, overnight.

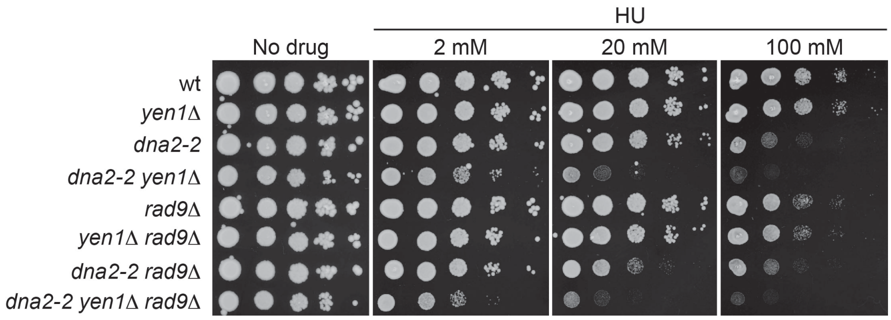

Supplementary Figure 5 │ Disruption of the DNA damage checkpoint does not suppress the sensitivity of Dna2 helicase-defective cells to chronic replication stress. Drop assays of the indicated strains on plates containing increasing amounts of HU, performed as described for Fig. 3.

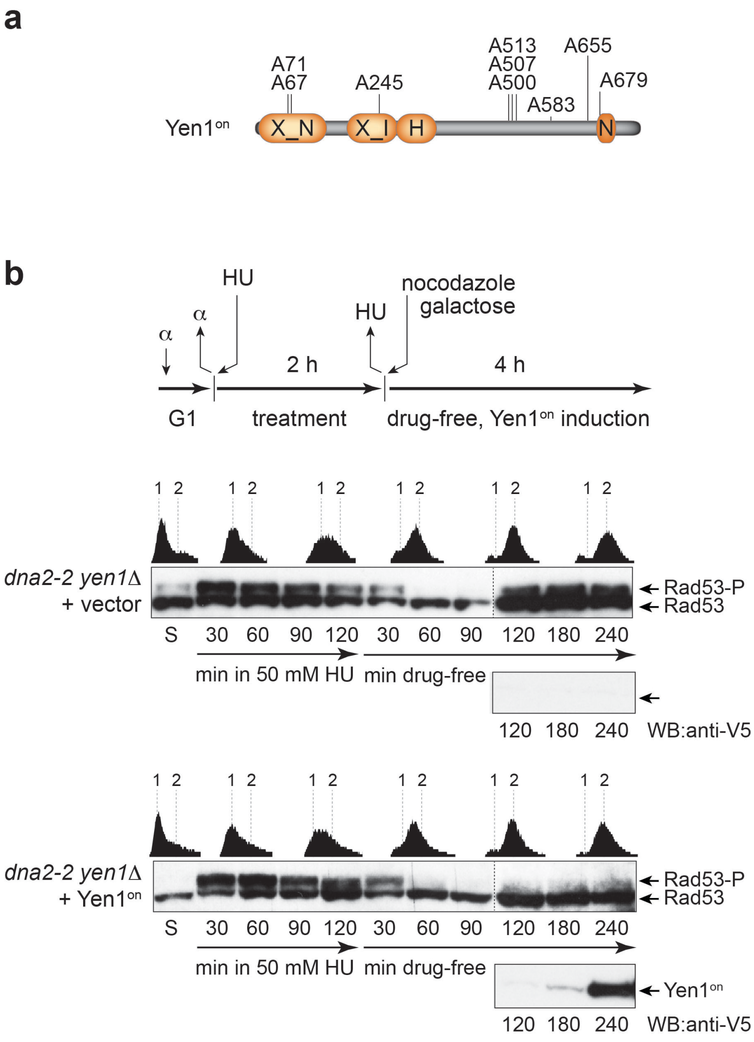

Supplementary Figure 6 │ Constitutively active Yen1^{on} suppresses post-replicative DNA damage checkpoint activation in Dna2 helicase-defective cells recovering from acute replication stress. (**a**) Yen1^{on} is mutated at all CDK consensus sites (serine to alanine substitutions), as indicated. This eliminates CDK-dependent control, allowing the active enzyme into the nucleus at all cell cycle stages. (**b**) Mitotic time-courses with *dna2-2 yen1*Δ cells harboring an empty vector control or a vector for the expression of YEN1^{on} under control of a galactose-inducible promoter. Cells were synchronized in G1, released into acute replication stress in the presence of 50 mM HU for 2 h, and then shifted to medium containing galactose to induce Yen1^{on} expression, and nocodazole to block cells in G2. Checkpoint activation and replication progression were monitored by assessing Rad53 phosphorylation (Rad53-P) and DNA content (1N and 2N indicated), respectively. Insets show the expression of Yen1^{on}, detected using an anti-V5 antibody.

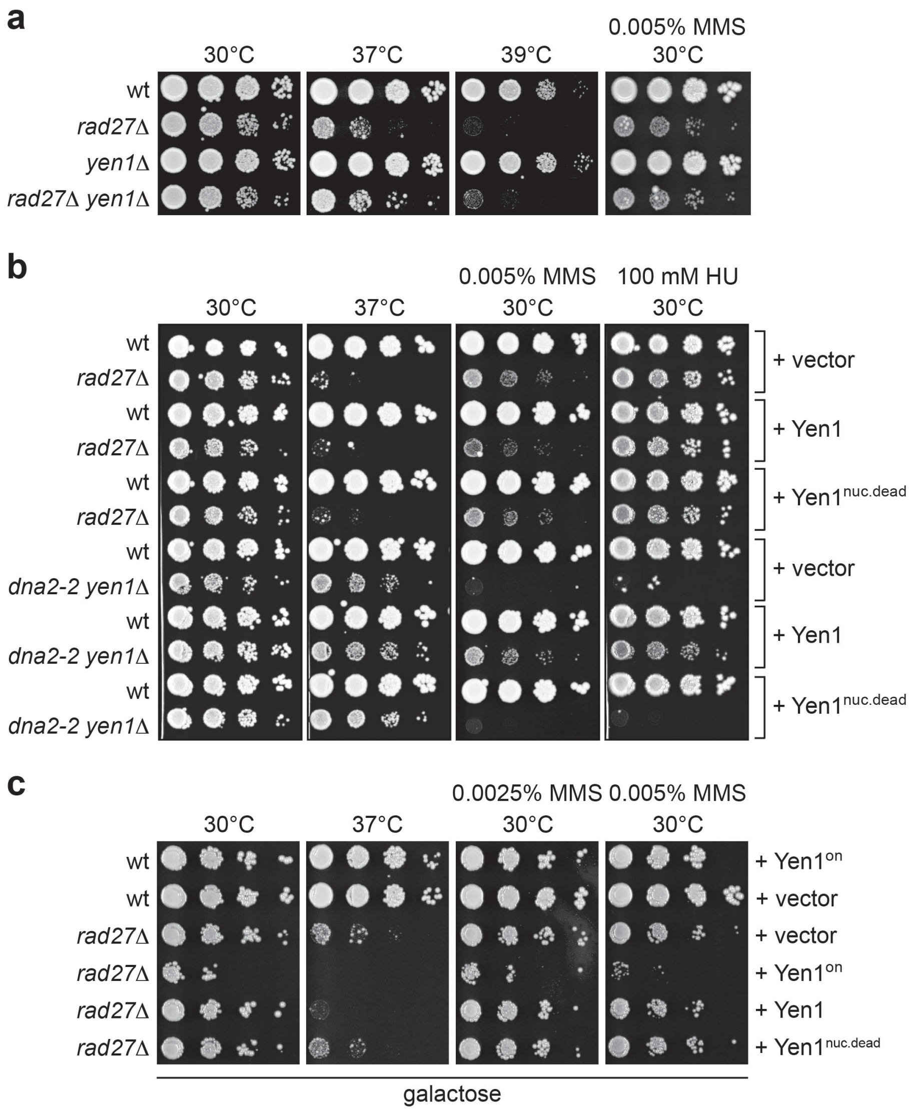

Supplementary Figure 7 │Genetic analysis of the relationship between Yen1 and Rad27. (**a**) A deletion of *YEN1* does not aggravate the temperature or MMS sensitivity of *rad27*Δ cells. (**b**) Constitutive or (**c**) galactose-induced expression of Yen1 or Yen1^{on} does not alleviate the temperature or MMS sensitivity of *rad27*Δ cells. Note how Yen1^{on} expression even attenuates growth in *rad27*Δ mutants under all conditions. In contrast, wild-type cells are inhibited by Yen1^{on} only in the presence of MMS. Drop assays were performed as described for Fig. 3 and plates were imaged after 2 to 3 days.

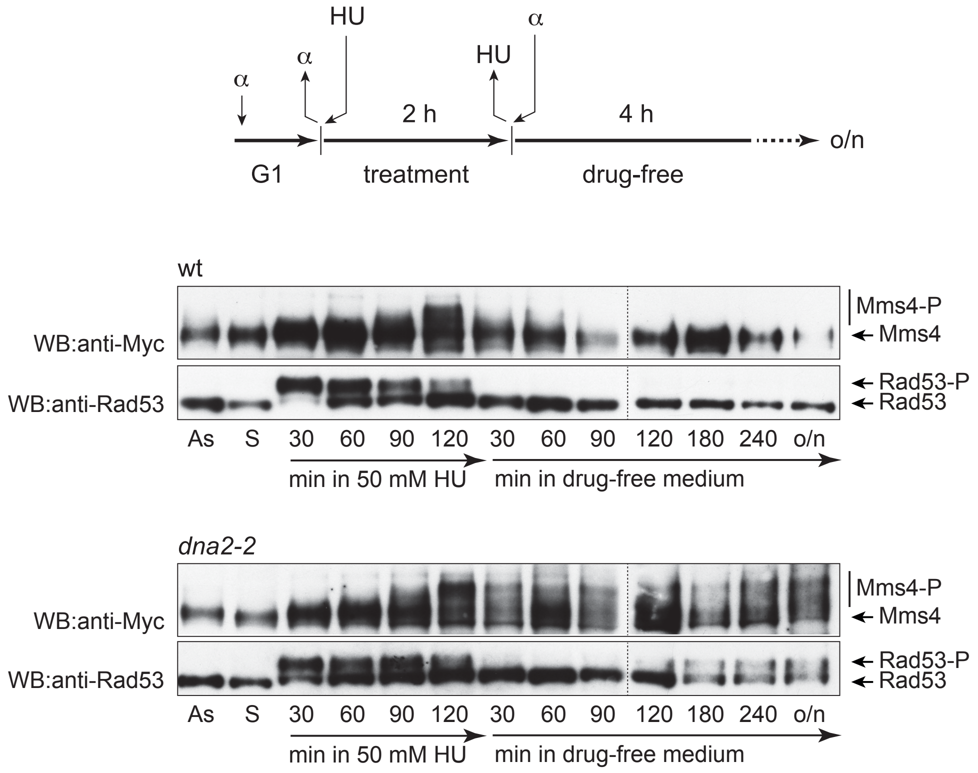

Supplementary Figure 8 │ Mus81-Mms4 is hyperphosphorylated in Dna2 helicase-defective cells after replication stress. Mitotic time-courses with wild-type and *dna2-2* cells with an endogenously 13 x Myc-tagged version of Mms4, performed as described for Fig. 4. Phosphorylation of Rad53 (Rad53-P) indicates checkpoint activation. Western blot analysis with an anti-Myc antibody reveals an upshift in the Mms4 signal caused by G2/M-specific hyperphosphorylation (Mms4-P). Hyperphosphorylation of Mms4 is transient in the wild-type, but persistent in *dna2-2* cells, which recover slowly from acute HU treatment and delay at the G2/M boundary (see also Fig. 4a). As, asynchronous; S, synchronous; o/n, overnight.

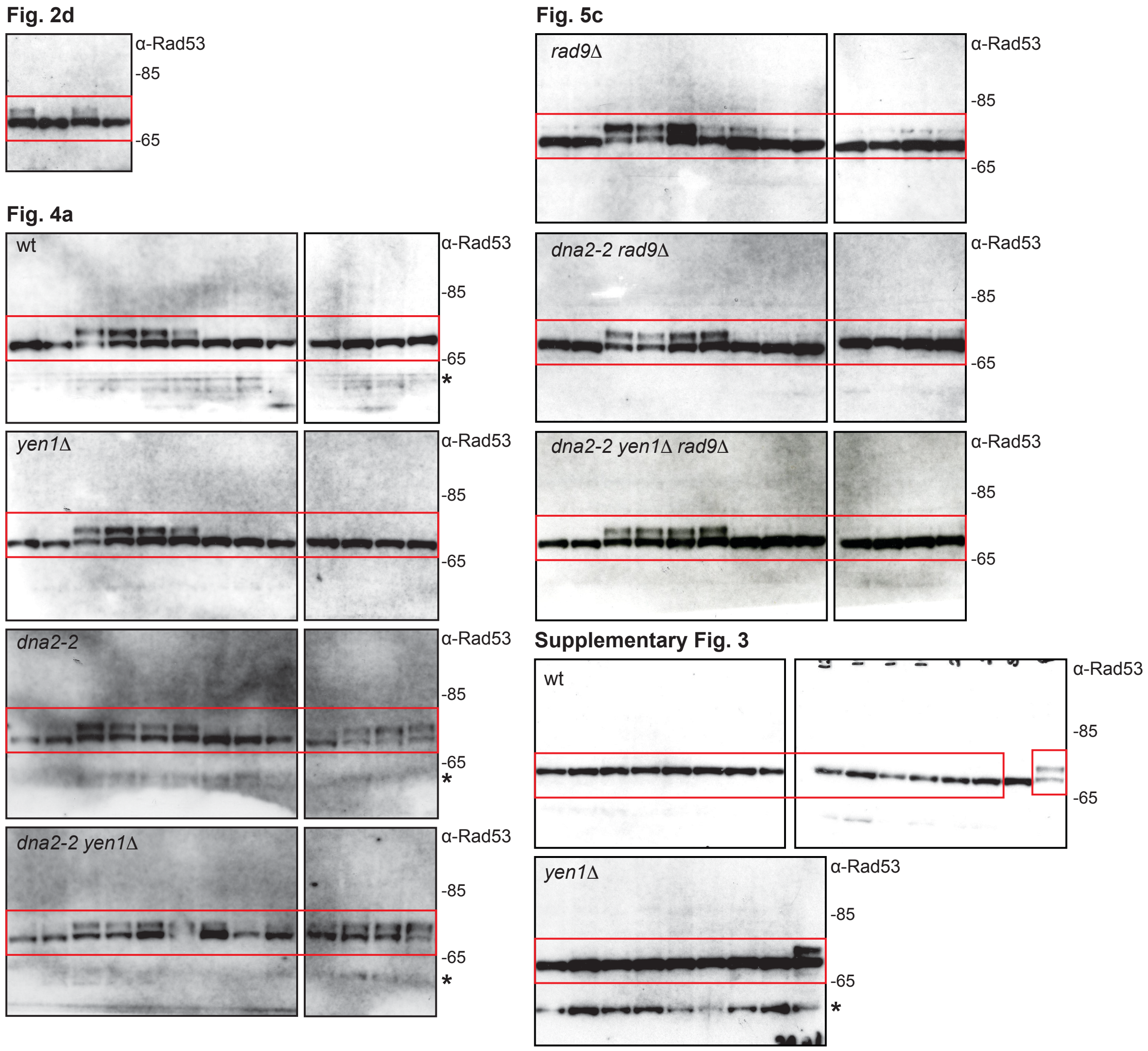

Supplementary Figure 9 │ Uncropped immunoblots. Boxed areas correspond to images presented in the indicated main text and supplementary figures. Size markers (kDa) are indicated. *, denotes unspecific bands.

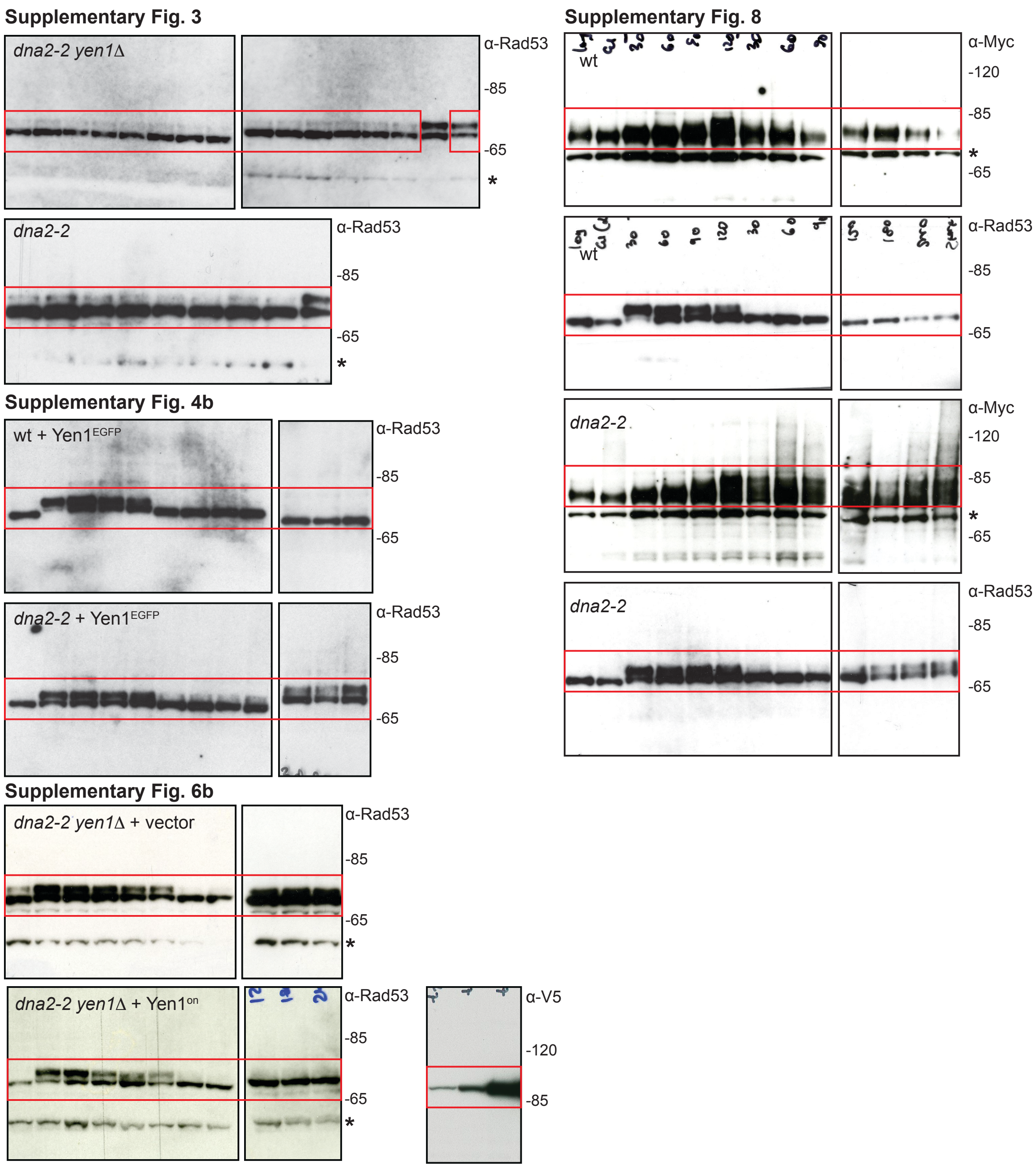

Supplementary Figure 9 │ Uncropped immunoblots. Boxed areas correspond to images presented in the indicated main text and supplementary figures. Size markers (kDa) are indicated. *, denotes unspecific bands.

Supplementary Tables

Supplementary Table 1 │ *S. cerevisiae* strains used in this study.

**Strain**	**Relevant genotype**	**Source**
BY4741 (wild-type)	*MAT***a** *his3Δ1 leu2Δ0 met15Δ0 ura3Δ0*	GE Healthcare
clone ID 174	BY4741 *yen1Δ*::*KanMX4*	GE Healthcare
clone ID 540	BY4741 *rad52Δ*::*KanMX4*	GE Healthcare
clone ID 3368	BY4741 *slx1Δ*::*KanMX4*	GE Healthcare
YRL31	BY4741 *DNA2-13myc:: KanMX4*	this study
YRL33	BY4741 *dna2-2-13myc:: KanMX4*	this study
YRL96	BY4741 *dna2-2*	this study
YRL97	BY4741 *dna2-2 yen1Δ*::*KanMX4*	this study
YRL98	BY4741 *dna2-2 mus81Δ*::*URA3*	this study
YRL99	BY4741 *dna2-2 slx1Δ*::*KanMX4*	this study
YRL129	BY4741 *dna2-2 rad52Δ*::*URA3*	this study
YRL133	BY4741 *rad9Δ*::*URA3*	this study
YRL134	BY4741 *yen1Δ*::*KanMX4 rad9Δ*::*URA3*	this study
YRL136	BY4741 *dna2-2 rad9Δ*::*URA3*	this study
YRL138	BY4741 *dna2-2 yen1Δ*::*KanMX4 rad9Δ*::*URA3*	this study
YRL241	BY4741 *MMS4-13myc::URA3*	this study
YRL243	BY4741 *dna2-2 MMS4-13myc::URA3*	this study
YRL249	BY4741 *rad27Δ*::*HIS3*	this study
YRL250	BY4741 *rad27Δ*::*HIS3 yen1Δ*::*KanMX4*	this study
YRL268	BY4741 *dna2-2 yen1Δ*::*KanMX4 rad52Δ*::*HIS3* p*DNA2* (*URA3*)	this study
YRL272	BY4741 *dna2-2 rad52Δ*::*HIS3* p*DNA2* (*URA3*)	this study
YWL169	BY4741 *mus81Δ*::*HIS3*	reference 1

Supplementary References

1. Blanco, M. G., Matos, J., Rass, U., Ip, S. C. Y. & West, S. C. Functional overlap between the structure-specific nucleases Yen1 and Mus81-Mms4 for DNA-damage repair in S. cerevisiae. *DNA Repair (Amst.)* **9,** 394–402 (2010).
